# Roles of transforming growth factor-β and phosphatidylinositol 3-kinase isoforms in integrin β1-mediated bio-behaviors of mouse lung telocytes

**DOI:** 10.1186/s12967-019-02181-2

**Published:** 2019-12-30

**Authors:** Dongli Song, Li Tang, Jianan Huang, Lu Wang, Tao Zeng, Xiangdong Wang

**Affiliations:** 1 Zhongshan Hospital Institute for Clinical Science, Shanghai Institute of Clinical Bioinformatics, Shanghai Engineering Research for AI Technology for Cardiopulmonary Diseases, Shanghai Medical College, Fudan University, Shanghai, China; 2 Key Laboratory of Systems Biology, Institute of Biochemistry and Cell Biology, Shanghai Institutes for Biological Sciences, Chinese Academy of Sciences, Shanghai, China

**Keywords:** Telocytes, Lung, PI3K, TGFβ, ITGB1

## Abstract

**Background:**

Telocytes (TCs) have the capacity of cell–cell communication with adjacent cells within the tissue, contributing to tissue repair and recovery from injury. The present study aims at investigating the molecular mechanisms by which the TGFβ1-ITGB1-PI3K signal pathways regulate TC cycle and proliferation.

**Methods:**

Gene expression of integrin (ITG) family were measured in mouse primary TCs to compare with other cells. TC proliferation, movement, cell cycle, and PI3K isoform protein genes were assayed in ITGB1-negative or positive mouse lung TCs treated with the inhibition of PI3Kp110α, PI3Kα/δ, PKCβ, or GSK3, followed by TGFβ1 treatment.

**Results:**

We found the characters and interactions of ITG or PKC family member networks in primary mouse lung TCs, different from other cells in the lung tissue. The deletion of ITGB1 changed TCs sensitivity to treatment with multifunctional cytokines or signal pathway inhibitors. The compensatory mechanisms occur among TGFβ1-induced PI3Kp110α, PI3Kα/δ, PKCβ, or GSK3 when ITGB1 gene was deleted, leading to alterations of TC cell cycle and proliferation. Of those PI3K isoform protein genes, mRNA expression of PIK3CG altered with ITGB1-negative TC cycle and proliferation.

**Conclusion:**

TCs have strong capacity of proliferation through the compensatory signaling mechanisms and contribute to the development of drug resistance due to alterations of TC sensitivity.

## Introduction

Telocytes (TCs) play an important role in cell–cell communication with adjacent cells within the tissue via direct patterns or indirect ways, such as cellular junctions, production of multi-factors and extracellular vesicles [1, 2]. TCs maintain local tissue homeostasis and cooperate with stem cells for organ repair and regeneration in diseases, inflammation, and fibrosis [3, 4]. However, mechanisms by which bio-behaviors of TCs are regulated are still unclear. Our recent study demonstrated that the transforming growth factor beta-1 (TGFβ1) interacted with phosphoinositide 3-kinase (PI3K) and regulated the proliferation and cell cycle phases of TCs [5]. Class IA PI3K is a heterodimer composed with two types of subunits, including one regulatory subunit and three catalytic subunits (p110α, p110β, p110δ). PI3K isoform proteins, e.g. PI3Kα/δ/β, PI3K/mTOR, and PI3K p110δ, contribute to the interactions and network functions of genes or proteins during TCs proliferation, especially PI3Kα/δ/β. TGFβ1 up-regulated the expression of *PIK3CA* coding p110α and *PIK3CB* coding p110β, while down-regulated the expression of *PIK3CD* coding p110δ and *PIK3CG* coding p110-γ in lung TCs [6]. PI3K p110α is involved in tumor growth, hypoxia, metastasis, or cell communication by increasing the tight junction formation [7] and the activity of glycogen synthase kinase-3 beta (GSK-3β) to promote cyclin D1 expression [8]. The present study furthermore investigates potential mechanisms of the interaction between TGFβ1 and PI3K isoforms in the regulation of TCs bio-behaviors.

PI3K/protein kinase B AKT/GSK3β signaling pathway-activated cell proliferation depends upon the alternations of TGFβ signaling by binding to integrins (ITG) [9–11]. TCs have the strong capacity of proliferation and of cell–cell communication with adjacent cells within the tissue, contributing to tissue repair and recovery from injury [6, 12]. The present study aims at investigating the molecular mechanisms by which the TGFβ1- integrin beta1 (ITGB1)-PI3K signal pathways regulate TCs cycle and proliferation. Gene expression profiles and special network characteristics of ITG family members were investigated among murine pulmonary TCs on days 5 (TC 5) and 10 (TC 10), fibroblasts, mesenchymal stem cells, alveolar type II cells (ATII), airway basal cells, proximal airway cells (PACs), CD8^+^ T cells come from bronchial lymph nodes (CD8 T BL), and CD8^+^ T cells from lung (CD8 T LL), respectively, like other genes [13]. Mouse lung TC Line was applied for investigating the patterns of PI3K catalytic isoform proteins or GSK3 and the regulation of TGF-β1 in TCs bio-behaviors were defined in mouse lung TCs [6]. We furthermore demonstrated effects of ITGB1 in PI3K catalytic isoform proteins or GSK3β-regulated mRNA expression of PI3K isoforms and defined the interactions among ITGB1, PI3K, and GSK3β in TCs bio-behaviors.

## Materials and methods

### Framework of the current study

We first analyzed the special network characteristics of ITG family molecules in primary lung TCs harvested from mice, as compared with alveolar type II cells, mesenchymal stem cells, airway epithelial cells, lymphocytes, and fibroblasts. After then mouse lung TCs was applied for investigating the patterns of PI3K catalytic isoform proteins (e.g. PI3K/p110α, PI3Kα/δ), Protein Kinase Cβ (PKCβ), or GSK3 in TCs proliferation, movement, differentiation and death. TGF-β1-regulated PI3K catalytic isoform proteins activity in TCs proliferation were validated in TCs with or without *ITGB1*. Effects of ITGB1 in PI3K catalytic isoform proteins or GSK3-regulated mRNA expression of PI3K isoforms were evaluated to define the interactions among ITGB1, PI3K, and GSK3.

### Network and molecular interactions of PKC family, GSK family or ITG family

Genomic probes were used for gene expression profiles analysis. 23,861 probes were contained in gene expression profiles of murine lung TCs, MSCs and Fbs, and 45,101 probes were included in ATII, ABCs, PACs, CD8 T-BL, and CD8 T-LL from GEO originally. Differentially expressed genes between two samples were identified through fold change filtering as reported previously [13]. Network and molecular interactions of *PKC* family, *GSK* family or *ITG* family were analyzed and figured according to the previous publication [14]. To reconstruct and show the state of gene network corresponding to each sample, we used differential network models (DEN) [14, 15] with the sample-specific network measurements [16]. The R package networkD3 (https://cran.r-project.org/web/packages/networkD3/index.html) was used to visualize the network structure of each sample, where the network nodes represent genes and network edges represent significant samples-specific edges or gene associations.

### Cell culture

This study was approved by the Fudan University Ethical Committee for animal experiments and mice were provided by Animal Facility in Biomedical Research Center of Zhongshan Hospital, Fudan University. Female BABL/c mice aged 6–8 weeks were used and the isolation of lung primary TCs were practiced as previous study [11]. After identification, lung primary TCs were transfected with SV40 large and small T antigen to constructed TCs. The telopodes were recorded and the expression of ckit, CD34, vimentin and PDGFR-α were detected for the identification of TCs maintaining the specific morphology and markers expression of TCs from generation 5 to generation 50 [6]. TCs were cultured in Dulbecco’s modified Eagle’s medium/F12 (DMEM/F12, GIBCO; Thermo Fisher Scientific, Inc.) supplemented with 10% fetal calf serum (FBS; GIBCO; Thermo Fisher Scientific, Inc.), 100 UI/ml penicillin, 0.1 mg/ml streptomycin (Sigma-Aldrich, St. Louis, USA), and cultured in a incubator, with 5% CO2 in air, at 37 °C. Cells were passaged when cell density were 60–70%. The cells were washed with PBS, digest with 0.25% trypsin, collection in new tubes and centrifuged at 400 × g for 5 min at room temperature. Then cells were seeded in new culture flasks.

### Lentivirus construction and infection

Lentivirus particles containing the *ITGB 1* shRNA sequence were constructed. The oligo (listed 5′–3′) includes *ITGB1 *shRNA forward: 5′-CCACAGAAGTTTACATTAA-3′and reverse: 5′- TTAATGTAAAGTTCTGTGG-3′; negative control forward: 5′-TTCTCCGAACGTGTCACGT-3′ and reverse: 5′-ACGAGACACGTTCGGAGAA-3′. TCs were seeded on 6-well plates with a density of 10^4^ cells/well and cultured for 24 h at 37 °C and replaced in fresh medium. Two μl of shRNA or negative control was added into each well and incubated for 12 h at 37 °C. Puromycin were added for screening. The cells were harvested for Real-time Quantitative PCR Detecting System (qPCR) analysis to determine the *ITGB 1* mRNA expression level after being cultured for 96 h at 37 °C. *ITGB1* knocked down TC cells were mentioned as TC^ITGB1−^ and TCs with* ITGB1* were named as TC^ITGB1+^.

### Immunofluorescent staining

Triple immunofluorescent staining for CD34/Vimentin/PDGFRα was used as previously reported [17, 18]. In brief, TCs were cultured on glass bottom cell culture dishes with 20 mm diameter glass (NEST, Nanjing, China) and were fixed in 4% paraformaldehyde containing 0.05% Triton-X-100 for 20 min. Then washed the dishes three times wash with 1 × PBS and blocked in 5% Bovine serum albumin (BSA) for 1 h. After incubated overnight at 4 °C with mouse anti-CD34 antibody, goat anti-vimentin antibody or rat anti- PDGFRα antibody (1:200 dilution; Abcam, Cambridge, UK) diluted in 1% bovine serum albumin (BSA) in PBS, the dishes were washing in PBS for three times. Then, dishes were incubated with APC conjugated anti-mouse secondary antibodies, PE conjugated anti-rat secondary antibodies and FITC conjugated anti-goat secondary antibodies (1:200 dilution; Jackson ImmunoResearch, USA). The nuclear were marked by DAPI according to the manufacture (KeyGEN BioTECH, Nanjing, China). Cells were observed and recorded under Olympus FV3000 Confocal Laser Scanning Microscope (DSS Imagetech Pvt. Ltd, New Delhi, India).

### Measurement of cell proliferation

TC^ITGB1+^ or TC^ITGB1−^ were digested and cultured in 96-well plates with the density of 5 × 10^3^ cells/well, 3–6 wells per group, followed by treatment with TGFβ1 at 0.5, 5.0, or 50 ng/ml, respectively, for 24 h to characterize TGFβ1-induced TCs proliferation. In order to evaluate roles of PI3K catalytic isoform proteins, 5 μM HS173 (PI3K p110α inhibitor, SelleckChem Co., Houston, USA), 5 μM GDC0941(PI3Kα/δ inhibitor, SelleckChem), 0.25 μM Enzastaurin (PKCβ inhibitor, SelleckChem) or 0.5 μM SB216763 (a potent and selective GSK-3 inhibitor for both GSK-3α and GSK-3β, SelleckChem)on TC^ITGB1+^ or TC^ITGB1−^ with or without TGFβ1 for 48 h. Ten microliters of CCK-8 reagents (Dojindo Molecular Technologies, Inc., Maryland, USA) was added to every well and incubated for 0.5 h at 37° C, with 5% CO_2_. We determined the absorbance at 450 nm using SpectraMax M5 Microplate Reader (Molecular Devices Instruments Inc., Sunnyvale, California, USA).

### RNA extraction and PCR

TC cells were seeded in 24-well plates with a density of 10^4^ cells/well and cultured for 24 h at 37 °C. Cells were starving for 12–24 h before stimulation. 0.05 ng/ml, 0.5 ng/ml or 5 ng/ml TGFβ1 for 24 h or 48 h were used for stimulation, and the PI3K inhibitors were added 2 h before TGFβ1 treatment. Cells were washed thrice with cold PBS, and total RNA was isolated and transcribed into single-stranded cDNA using the 1st Strand cDNA Synthesis Kit (AMV, Roche Molecular Systems, Inc., Branchburg, USA) for Reverse Transcription-Polymerase Chain Reaction (RT-PCR) following the recommendations of the manufacturer. cDNA was synthesized from 1 µg of total RNA using PrimeScript^®^ RT reagent Kit (Takara Bio Inc., Shiga, Japan). PCR was performed with 1 µl of cDNA using GoTaq polymerase (Promega Corporation, Madison, USA) for 25 cycles with specific primers for genes* ITGB1* forward primer: GTCTTGGAACGGATTTGATGA and reverse: TTTGCTGGGGTTGTGCTAAT; GAPDH forward: CGGAGTCAACGGATTTGGTCGTAT and reverse: AGCCTTCTCCATGGTGGTGAAGAC. PCR reaction products were resolved through a 0.8% agarose gel in 1 × TAE and stained with Gelred (Biotium Inc., Newark, USA).

### Detection of cell bio-behaviors

TCs were treated with 0.05 μM, 0.5 μM or 5 μM HS173 (PI3K/p110α inhibitor), GDC0941(PI3Kα/δ inhibitor), or 0.5 μM SB216763 (a potent and selective GSK-3 inhibitor for both GSK-3α and GSK-3β) or 0.025 μM, 0.25 μM or 2.5 μM Enzastaurin (PKCβ inhibitor)with or without TGFβ1 for 48 h. The bio-behaviors of TCs were recorded and analyzed using a Cell-IQ cell culturing platform (Chip-Man Technologies, Tampere, Finland), equipped with a phase-contrast microscope (Nikon CFI Achromat phase contrast objective with 10 magnification) and a camera [12]. These bio-behaviors, including total cell number, cell morphology, and cell movement, can be monitored and recorded as time-lapse data by this Cell-IQ system uses machine vision technology. Images were captured at about 30 min intervals for 1 week. Analysis was carried out with a freely distributed Image software (McMaster Biophotonics Facility, Hamilton, ON), using the Manual Tracking plugin created by Fabrice Cordelie´res (Institute Curie, Orsay, France).

### Cell cycle assay

PI staining was used for cell cycle analysis of primary TCs as described in manufacturer. In brief, cells were collected and fixed in 75% ethanol at 4 °C for overnight. After centrifuged and washed, 0.5 ml PI/RNase Staining Buffer (BD Pharmingen, NJ, USA) was added to each tube for 15 min at room temperature. Samples were examined with a fluorescence-activated cell sorting flow cytometer, BD FACS Aria II (Becton, Dickinson and Company, NJ, USA) and DNA histograms were analyzed with Flowjo 7.6.1 software. Each test was repeated in triplicate.

### Statistics

Data were analyzed using SPSS Statistics 20 (IBM, Chicago, USA). Statistical differences between two groups were compared by *t* test. Statistical differences among more than two groups were determined using ANOVA. Each point corresponds to the mean ± S.E.M. and p < 0.05 was considered significant.

## Results

**Fig. 1:**
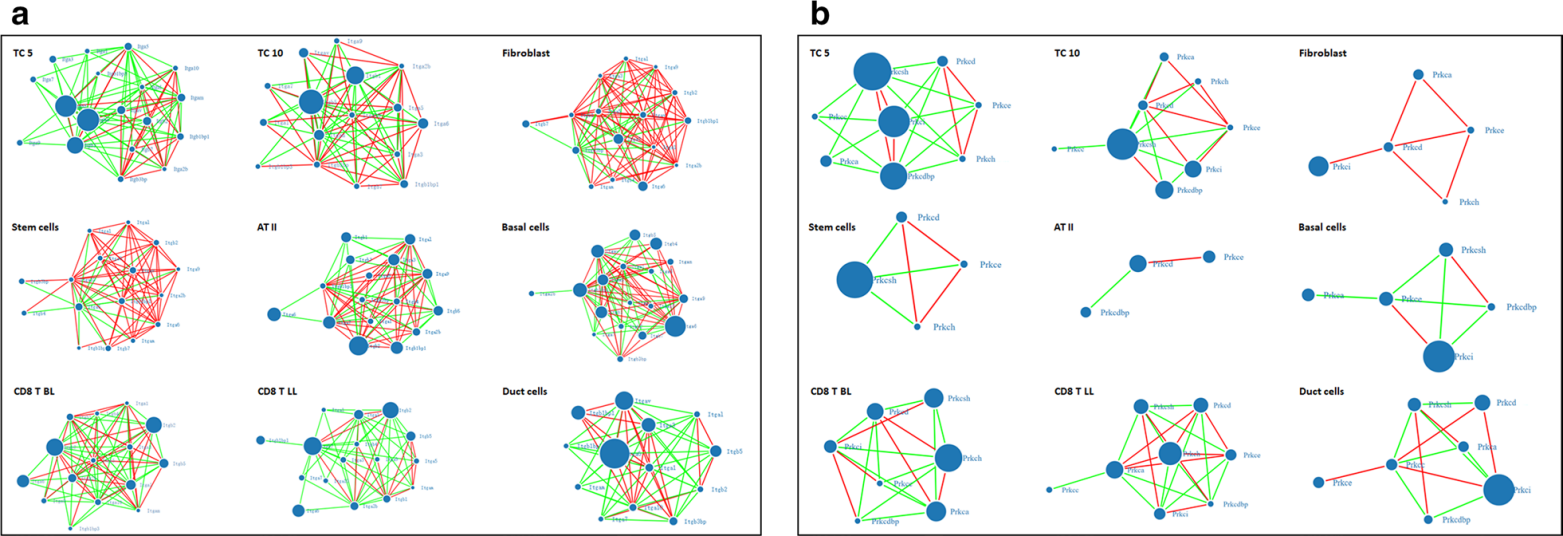
The characters of ITGB1, and PKC networks and interactions in mouse lung primary TCs cultured for 5 days (TC 5) and 10 days (TC 10) were compared with other tissue cells, e.g. fibroblasts, mesenchymal stem cells (Stem cells), alveolar type II cells (ATII), airway basal cells (Basal cells), proximal airway cells (Duct cells), CD8^+^ T cells come from bronchial lymph nodes (CD8 T BL), and CD8^+^ T cells from lung (CD8 T LL). **a** Network characters and molecular interactions of ITG family genes; **b** Network characters and molecular interactions of PKC family genes and its receptor family genes

Figure 1 demonstrated the specificity of PKC, GSK3 and ITG family in TCs, network characters and molecular interactions of PKC family genes, GSK3 family genes or ITG family genes. Network elements and interactions of ITG subunit genes in mouse lung primary TCs were changed from day 5 to day 10 after the culture, and had positive communication (Fig. 1a). The network characters and interactions of PKC genes in TCs were different from those in fibroblasts, mesenchymal stem cells, ATII, airway basal cells, PACs, and varied significantly between TCs on days 5 and 10 (Fig. 1b). The network characters of GSK subunit genes had negative communication on day 5, and became positive links on day 10 (Additional file 1: Figure S1).

**Fig. 2:**
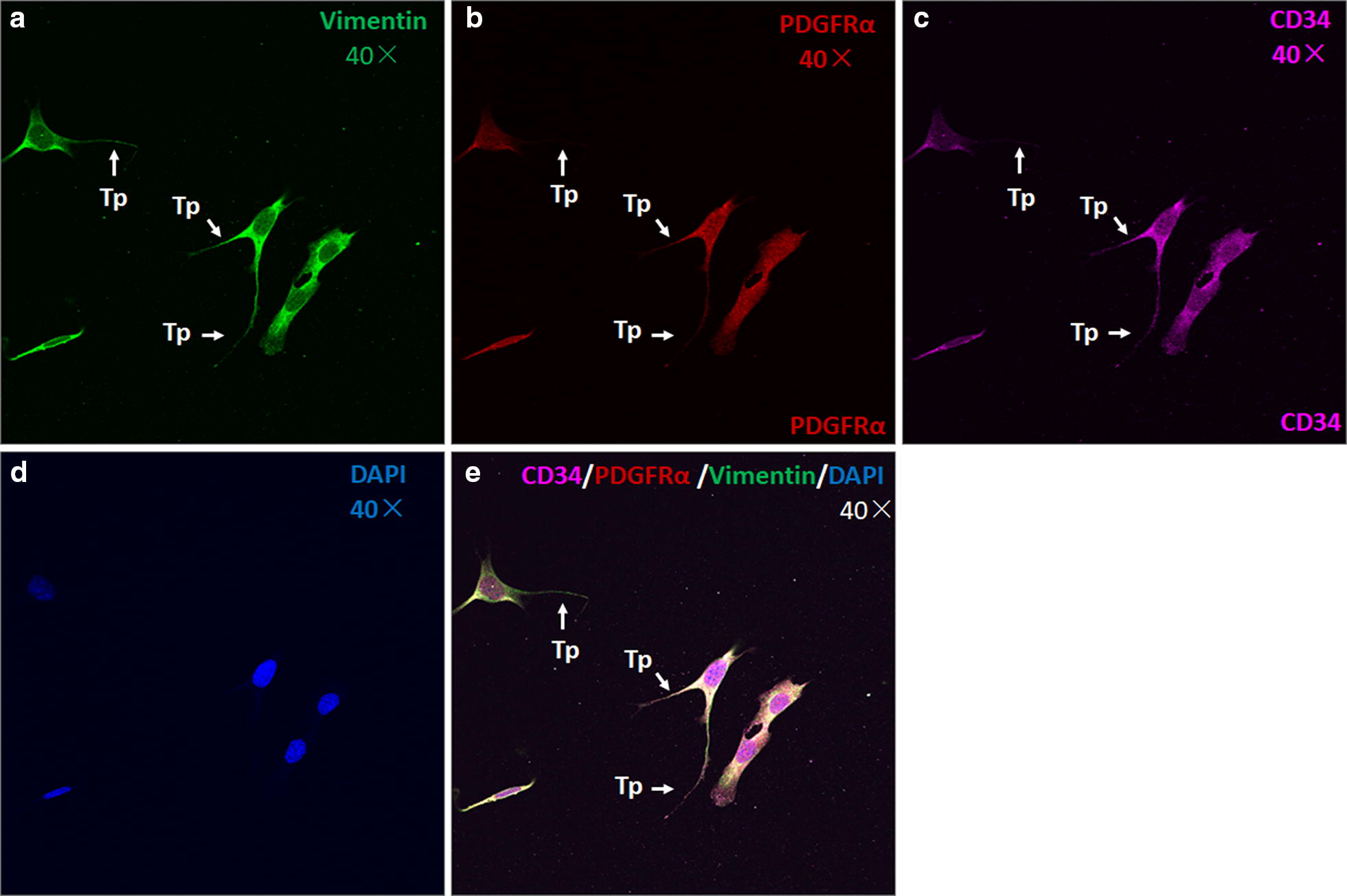
TCs are CD34/PDGFR-α/vimentin triple positive in vitro. Triple immunofluorescence labelling for vimentin (**a**, green), PDGFR-α (**b**, red) and CD34 (**c**, purple) with DAPI (**d**, blue) counterstain for nuclei. TCs are CD34, PDGFR-α and vimentin positive (**e**). Arrows show typical TCs with long and thin telopodes (Tp) with dilations. Original magnification ×400

**Fig. 3:**
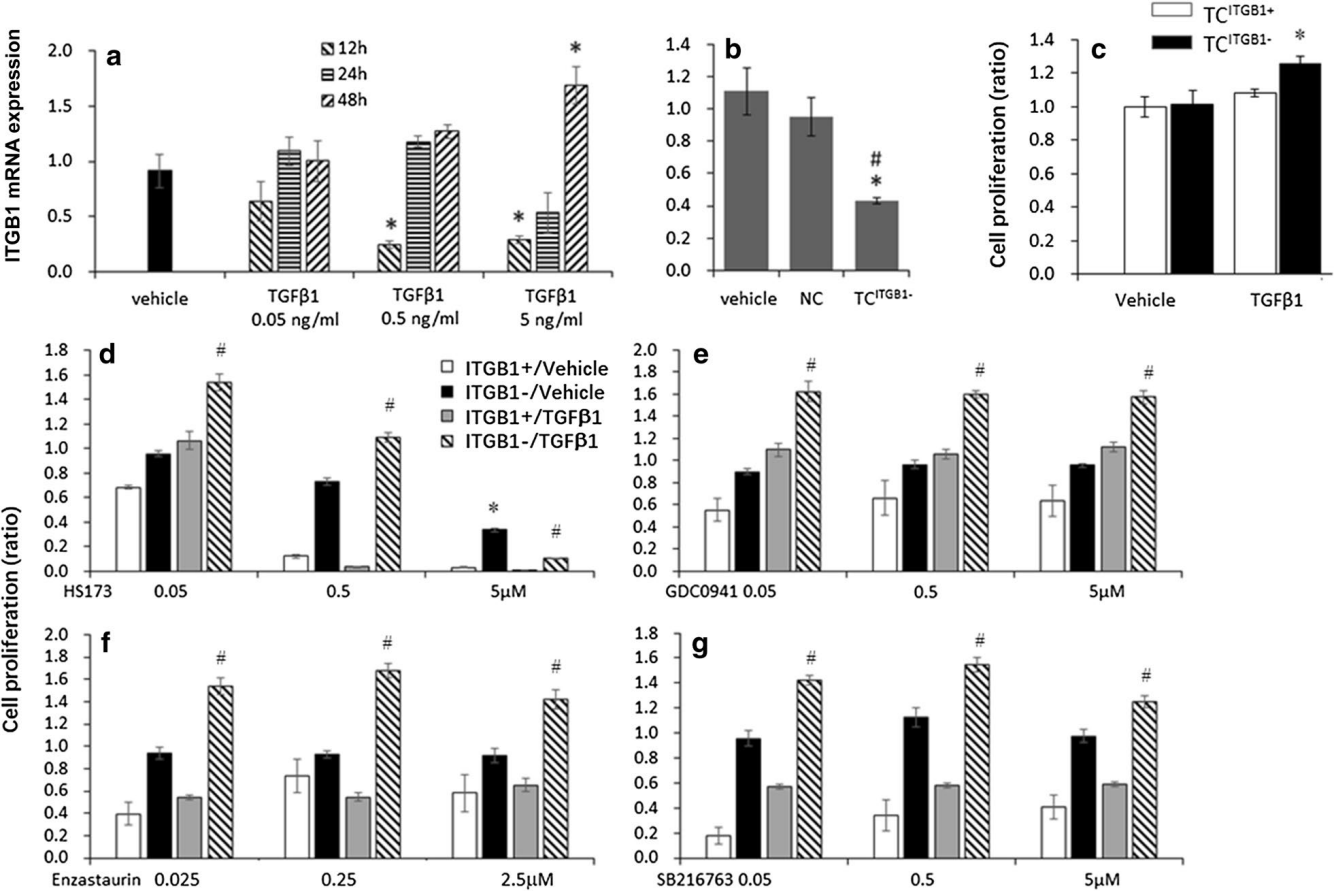
The effects of ITGB1 on cell proliferation of TCs treated with PI3K/p110α, PI3Kα/δ, PKCβ, or GSK3 inhibitors. **a** Expression of ITGB1 mRNA in TCs 12, 24, and 48 h after treatment with TGFβ1 at 0.05, 0.5, or 5 ng/ml, respectively. **b** Expression of ITGB1 mRNA in TCs treated with vehicle, non-specific sequencing (NC), or ITGB1 siRNA (TC^ITGB1−^), * stand for *p* values less than 0.05, as compared with TCs; ^*#*^ stand for p values less than 0.05, respectively, as compared with NC. **c** Proliferation of TCs treated with vehicle (TC^ITGB1+^) or ITGB1 siRNA (TC^ITGB1−^), *stand for *p* values less than 0.05, as compared with TC^ITGB1+^ treated with TGFβ1. **d** Proliferation of TGFβ1 or vehicle-treated TC^ITGB1+^ or TC^ITGB1−^ 24 h after treatment with HS173 (PI3Kp110α inhibitor) at 0.05, 0.5 or 5 μM. **e** Proliferation of TGFβ1 or vehicle-treated TC^ITGB1+^ or TC^ITGB1−^ after treatment with GDC0941 (PI3Kα/δ inhibitor) at 0.05, 0.5 or 5 μM. **f** Proliferation of TGFβ1 or vehicle-treated TC^ITGB1+^ or TC^ITGB1−^ after treatment with Enzastaurin (PKCβ inhibitor) at 0.025, 0.25 or 2.5 μM. **g** Proliferation of TGFβ1 or vehicle-treated TC^ITGB1+^ or TC^ITGB1−^ after treatment with SB216763 (GSK3 inhibitor). n = 3–6, *stand for *p* values less than 0.05, respectively, as compared with TC^ITGB1+^; ^*#*^ stand for *p* values less than 0.05, respectively, as compared with TC^ITGB1+^ treated with TGFβ1

**Fig. 4:**
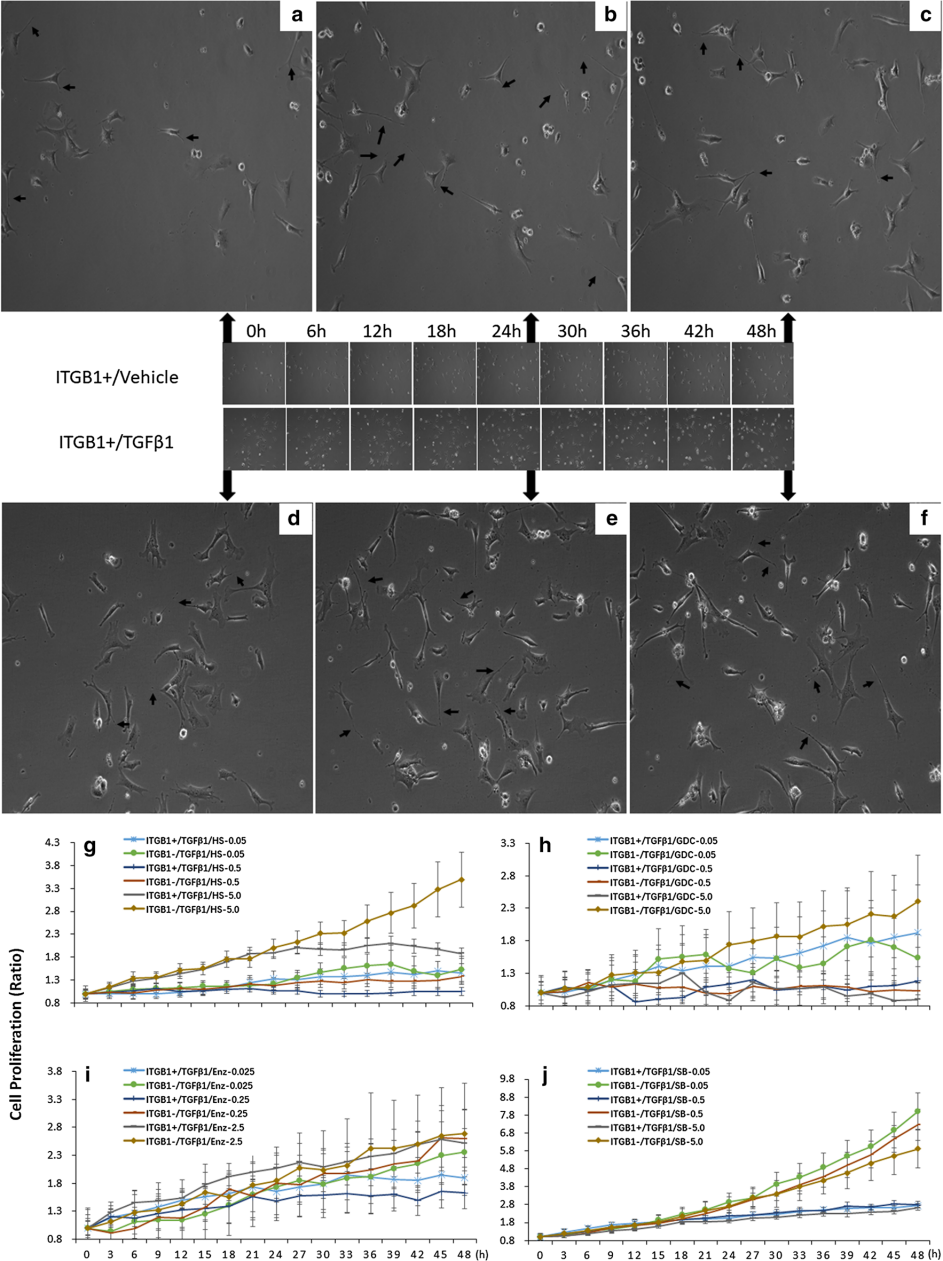
The effects of ITGB1on the proliferation curve of TC ^ITGB1+^ treated with PI3K inhibitors and TGFβ1. **a**–**c** Representative photos of TCs cultured for 0 h, 24 h and 48 h captured by celliq, respectively. **d**–**f** representative photos of TCs treated within TGFβ1 for 0 h, 24 h and 48 h, respectively. Arrows show typical TCs with long and thin telopodes with dilations. Original magnification ×100. **g** Analysis of TC ^ITGB1+^ or TC^ITGB1−^ proliferation with the treatment of HS173 and TGFβ1. **h** Analysis of TC ^ITGB1+^ or TC^ITGB1−^ proliferation with the treatment of GDC0941 and TGFβ1. **i** Analysis of TC ^ITGB1+^ or TC^ITGB1−^ proliferation with the treatment of Enzastaurin and TGFβ1. **j** Analysis of TC ^ITGB1+^ or TC^ITGB1−^ proliferation with the treatment of SB216763 and TGFβ1, n = 6–8, **stand for *p* values less than 0.05, as compared with TC ^ITGB1+^ treated with TGFβ1

Mouse lung TCs were successfully isolated and constructed as a cell line as our previous reported [6]. These TCs have special characteristics including relatively small cell body and very long and thin Tps with lots of dilations (Fig. 4a). To further confirm that the cells were TCs, triple immunofluorescent staining for CD34/PDGFR-α/vimentin was used. We found that these cells were triple positive for CD34/PDGFR-α/vimentin (Fig. 2), indicating that these cells were TCs. To understand potential regulations between those factors in TCs, we firstly investigated effects of external TGFβ1 at different concentrations and time points on TCs with ITGB1 (TC^ITGB1+^) and selected the concentration of TGFβ1 at 5 ng/ml for 48 h as the condition of further studies (Fig. 3a). ITGB1 knocked down TC cells (TC^ITGB1−^) were generated by lentivirus construction and infection with ITGB1 shRNA and evaluated by expression of ITGB1 gene (Fig. 3b). TGFβ1 could induced the increased cell proliferations in TC^ITGB1−^ cells at 48 h, as compared to TC^ITGB1+^ cells (Fig. 3c). HS173 inhibited cell proliferation significantly in a dose-dependent pattern in TC^ITGB1+^ cells with or without TGFβ1 administration and had higher responses about 98% inhibitory rate at 5 μM, as compared to TC^ITGB1−^ cells (Fig. 3d). The proliferation of TGFβ1-pretreated TC^ITGB1−^ cells was higher than vehicle-pretreated ones after HS173 administration, especially at doses of 0.05 and 0.5 μM. The cell proliferation reduced from the low dose of GDC0941 (0.05 μM, Fig. 3e), Enzastaurin (0.025 μM, Fig. 3f), or SB216763 (0.05 μM, Fig. 3g), rather than in TC^ITGB1−^ cells. TGFβ1-pretreated TC^ITGB1−^ cells had significantly higher cell proliferation than vehicle-pretreated TC^ITGB1−^ cells, even higher than TC^ITGB1+^ cells.

**Fig. 5:**
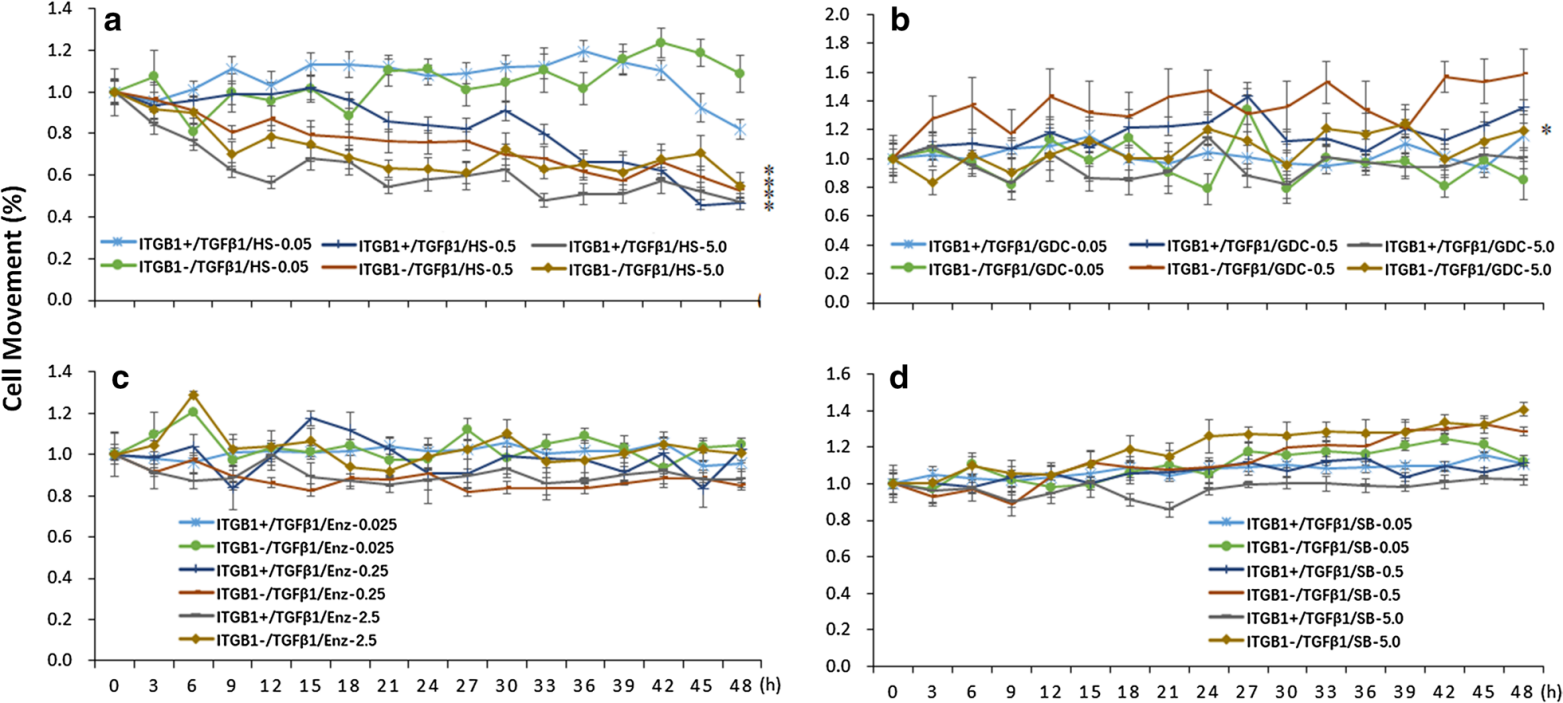
The effects of ITGB1on the movement curve of TCs treated with PI3K inhibitors and TGFβ1. **a** Analysis of TC ^ITGB1+^ or TC^ITGB1−^ movement with the treatment of HS173 and TGFβ1. **b** Analysis of TC^ITGB1+^ or TC^ITGB1−^ movement with the treatment of GDC0941 and TGFβ1. **c** Analysis of TC^ITGB1+^ or TC^ITGB1−^ movement with the treatment of Enzastaurin and TGFβ1. **d** Analysis of TC^ITGB1+^ or TC^ITGB1−^ movement with the treatment of SB216763 and TGFβ1, n = 6–8, *stand for *p* values less than 0.05, as compared with TC ^ITGB1+^

We furthermore investigated dynamic sensitivities of TCs with or without ITGB1 gene to TGFβ1 and found that TGFβ1 reduced dynamic proliferation of TCs as compared to vehicle, and TC^ITGB1−^ cells had lower proliferation than TC^ITGB1+^ cells during TGFβ1 challenge (Additional file 2: Figure S2A). The morphology of TC ^ITGB1+^ treat with or without TGFβ1 for 24 h and 48 h were shown with Tps in Fig. 4a–f. TC^ITGB1−^ cells had higher levels of cell proliferation in co-existence of TGFβ1 with HS173 at different doses with a dose-dependent pattern (Fig. 4g), in TGFβ1 with GDC0941 at 5.0 μM (Fig. 4h), in TGFβ1 with Enzastaurin at 0.25 μM (Fig. 4i), or in TGFβ1 with SB216763 at three doses (Fig. 4j). Of those TGFβ1-treated TCs, cell proliferation was lower after the administration of GDC0941 at 0.5 μM than at 0.05 μM. There was no significant difference of the dynamic movement capacity among TCs and TC^ITGB1−^ cells with or without TGFβ1 challenge (Additional file 2: Figure S2B). HS173 at 0.5 and 5.0 μM reduced the movement of TC^ITGB1+^ or TC^ITGB1−^ cells in co-existence of TGFβ1 (Fig. 5a). GDC0941 at 5.0 μM increased the movement of TC^ITGB1+^ or TC^ITGB1−^ cells, between which TC^ITGB1−^ cells movement was higher during TGFβ1 challenge (Fig. 5b). Cell movement of TCs with or without ITGB1 gene did not show significantly different during the co-existence of TGFβ1 with Enzastaurin (Fig. 5c) or SB216763 (Fig. 5d), although TC^ITGB1−^ cells treated with TGFβ1 and SB216763 showed slightly high movement.

**Fig. 6:**
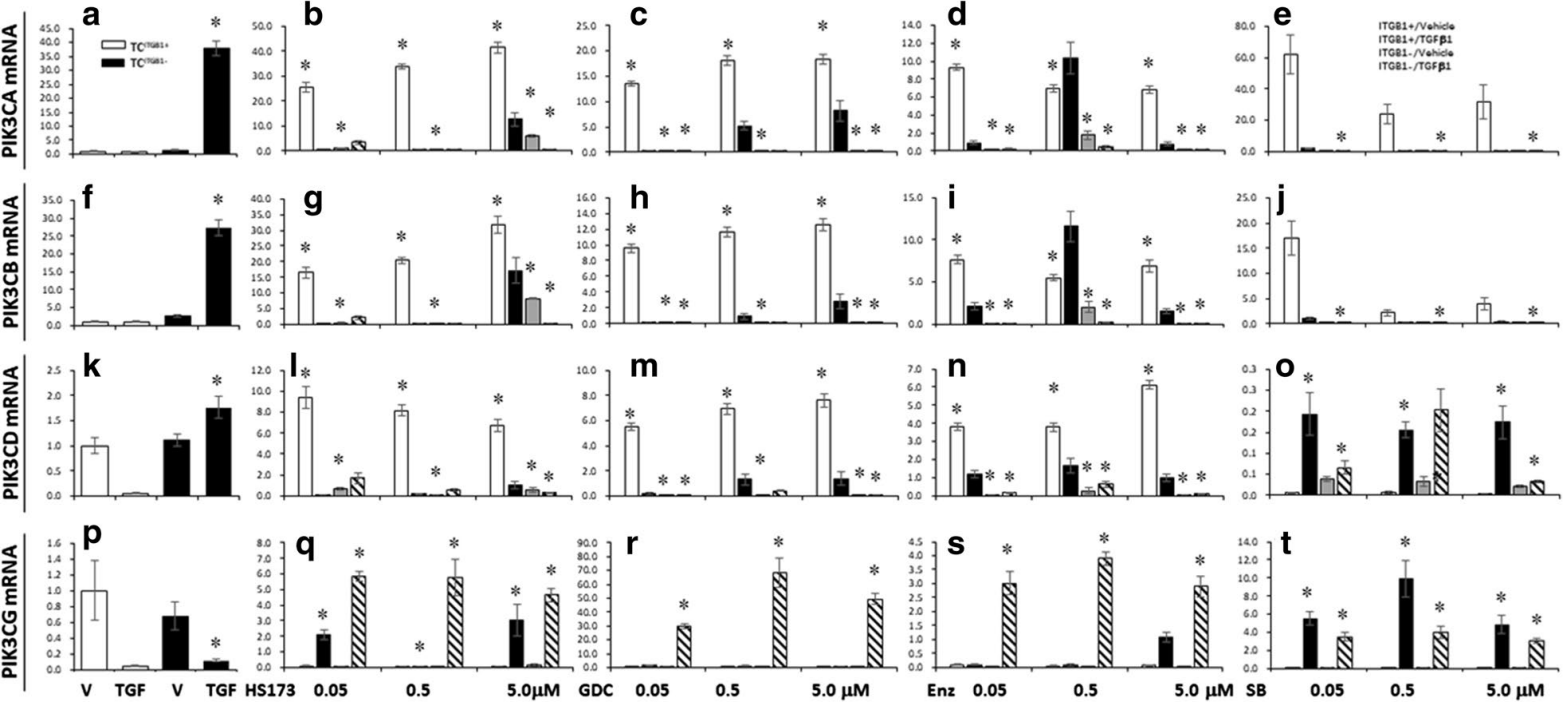
Analysis of mRNA levels of *PIK3CA*, *PIK3CB*, *PIK3CG*, or *PIK3CD* in TC^ITGB1+^or TC^ITGB1−^ treated with PI3K inhibitors and/or stimulated with TGFβ1. **a**–**e** mRNA levels of *PIK3CA*, **b**–**j** mRNA levels of *PIK3CB,***k**–**o** mRNA levels of *PIK3CD*, **p**–**t** mRNA levels of *PIK3CG* monitored after TC^ITGB1+^or TC^ITGB1−^ co-treated with TGFβ1 and PI3Kp110α, PI3Kα/δ, PKCβ, GSK3 inhibitors, respectively, n = 3–6, *stand for *p* values less than 0.05, as compared with TC ^ITGB1+^

In order to define roles of ITGB1 in the expression of PIK3 class 1 catalytic genes, *PIK3CA* (coding PI3K p110α), *PIK3CB* (coding PI3K p110β), *PIK3CD* (coding PI3K p110δ), and *PIK3CG* genes (coding PI3K p110γ) were monitored after TCs were co-treated with TGFβ1 and pan-PI3K, PI3K p110α, PKC, GSK3β inhibitors. We found that TGFβ1 increased expression of *PIK3CA* (Fig. 6a), *PIK3CB* (Fig. 6f), *PIK3CD* (Fig. 6k), *PIK3CG* (Fig. 6p) in TC^ITGB1−^ cells, respectively. mRNA levels of *PIK3CA*, *PIK3CB*, and PIK3CD were significantly higher in TC^ITGB1+^ treated with HS173 (Fig. 6b, g, l, q), GDC0941 (Fig. 6c, h, m, r), or Enzastaurin (Fig. 6d, i, n, s), as compared to other groups (p < 0.05 or less, respectively). We also noticed that mRNA levels of PIK3CA, PIK3CB, and PIK3CD were lower in TC^ITGB1−^ cells treated with HS173 at 5 μM as compared to the corresponding TC^ITGB1+^ with vehicle, and in TGFβ1-treated cells after HS173 at 5.0 μM, GDC0941 at 0.5 and 5.0 μM, Enzastaurin at all doses, or SB216763 as compared to vehicle-treated cells. mRNA expression of *PIK3CA* and *PIK3CB* had similar patterns of changes. mRNA of *PIK3CD* was lower in TC^ITGB1−^ treated with SB216763 (Fig. 6o). mRNA of *PIK3CG* was higher in TC^ITGB1−^ treated with SB216763 (Fig. 6o) in TC^ITGB1−^ with HS173 (Fig. 6q), GDC0941(Fig. 6r), Enzastaurin (Fig. 6s), and SB216763(Fig. 6t). An increase in cell differentiation was noted in TC^ITGB1−^ treated with TGFβ1 and HS173 (Additional file 3: Figure S3), while the cell death number was significantly lower in TC^ITGB1−^ (vs TC^ITGB1+^) treated with TGFβ1 and HS173 (Additional file 4: Figure S4A) or with TGFβ1 and Enzastaurin (Additional file 4: Figure S4C), but significantly higher in TC^ITGB1−^ (vs TC^ITGB1+^) treated with TGFβ1 and SB216763 (Additional file 1: Figure S4D). Relevant morphological data was recorded and shown in Figure 4 A-F and Additional file 5: Figure S5.

**Fig. 7:**
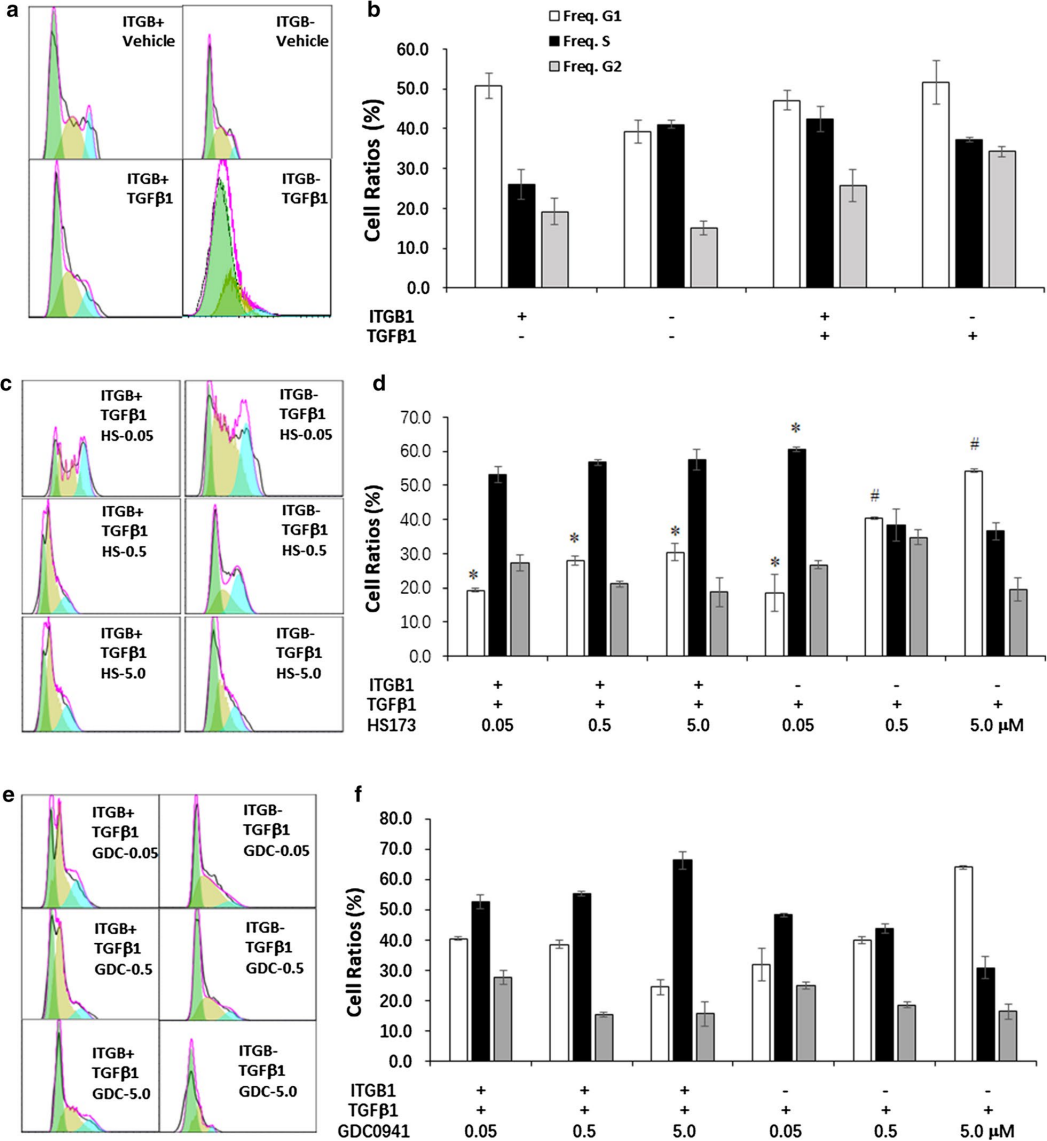

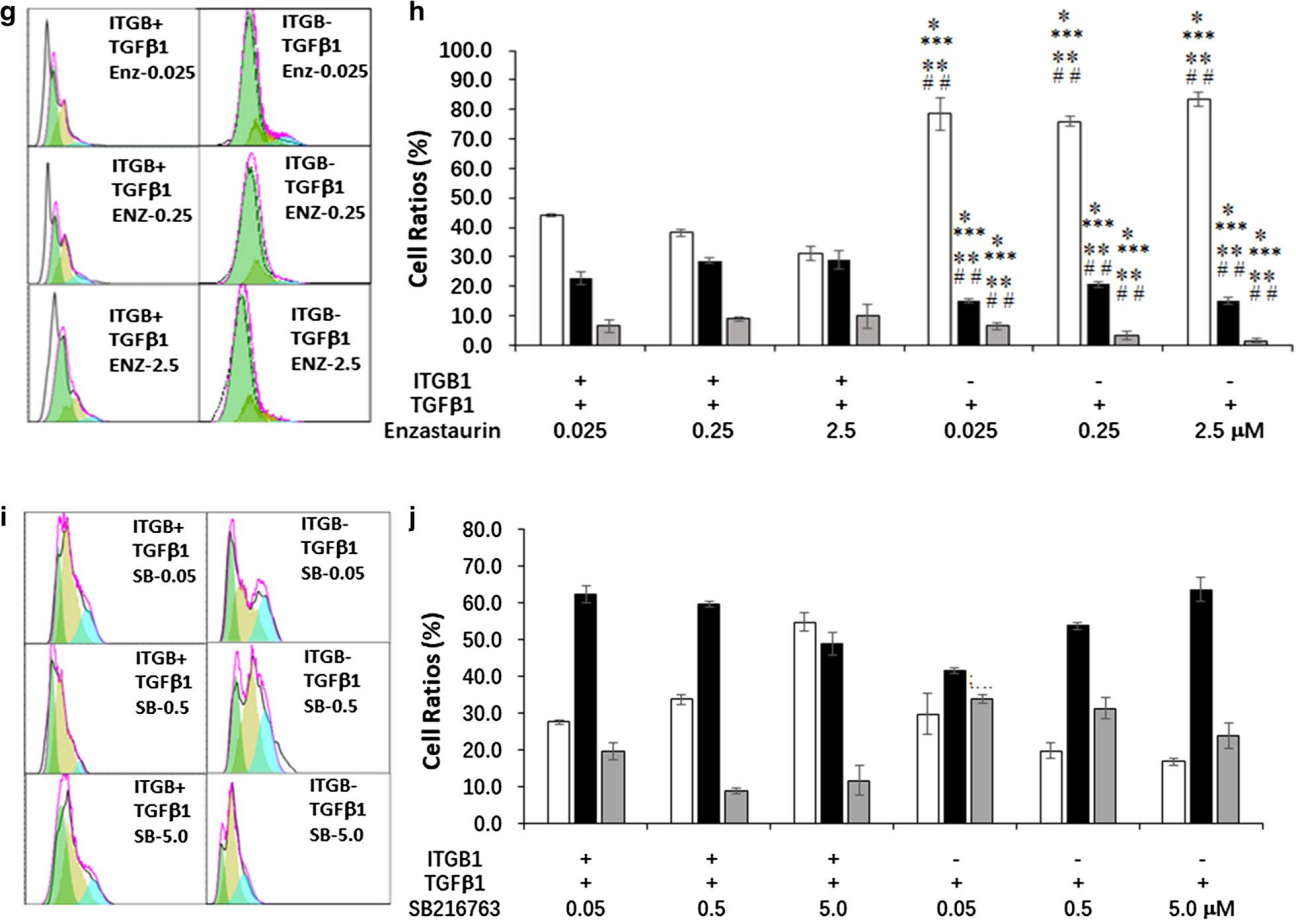
The effects of ITGB1 on cell cycle of TCs treated with PI3K inhibitors and TGFβ1. **a**–**d** Cell cycle analysis of HS173 (PI3K/p110α inhibitor) in TGFβ1-induced TC^ITGB1+^ by flowcytometry. **e**–**f**. Cell cycle analysis of GDC0941 (pan-PI3K inhibitor) in TGFβ1-induced TC^ITGB1+^ by flowcytometry. **g**–**h** Cell cycle analysis of Enzastaurin (PI3K/PKC inhibitor) in TGFβ1-induced TC^ITGB1+^ by flowcytometry. **i**–**j** Cell cycle analysis of SB216763 (GSK-3 inhibitor) in TGFβ1-induced TC^ITGB1+^ by flowcytometry, n = 3–6, * stand for *p* values less than 0.05, as compared with TGFβ1-induced TC ^ITGB1+^, ^*#*^ stand for *p* values less than 0.05, as TC^ITGB1−^ treated with PI3K inhibitors and TGFβ1compared with TC ^ITGB1+^ treated with PI3K inhibitors and TGFβ1. **   stand for *p* values less than 0.05, as compared with TC ^ITGB1+^, ^*##*^  stand for *p* values less than 0.05, as compared with TC ^ITGB1−^, ***stand for *p* values less than 0.05, as compared with  TGFβ1-induced TC ^ITGB1+^

We further analyzed the cell cycle of TC ^ITGB1+^ and TCs^ITGB1−^ to find out the mechanisms in ITGB1 mediated TCs proliferation (Fig. 7a, b) alteration and found that TGFβ1-treated TC ^ITGB1+^ or TC^ITGB1−^ number in G1 phase increased in a dose-dependent patter of HS173, while still was lower than after TGFβ1 and vehicle, except for HS173 at 5.0 μM (Fig. 7c, d). TC^ITGB1−^ number in G1 was significantly higher than TC ^ITGB1+^ after co-treated with TGFβ1 and HS173. HS173 at 0.05 μM decreased the ratios of G1 phase and induced S phase arrest in TC^ITGB1−^ compared with TC ^ITGB1+^ which decreased in G1 and increased in S phase after treated with TGFβ1 and HS173(Fig. 7c, d). TC ^ITGB1+^ decreased while TC^ITGB1−^ increased in G2 phase with a dose-dependent pattern after TGFβ1 and GDC0941 (Fig. 7e, f). In opposite, TC^ITGB1−^ decreased while TCs increased in S and G2 phases. TC^ITGB1−^ number increased in G1 phase and deceased in S and G2 phases after treatment with Enzastaurin, as compared with TC ^ITGB1+^ or TC^ITGB1−^ with or without TGFβ1 (Fig. 7g, h). TC ^ITGB1+^ increased in G1 phase and TC^ITGB1−^ decreased in G1 and G2 phases gradually with increased doses of SB216763 after treated with TGFβ1 and SB216763 (Fig. 7i, j), where the number of TC^ITGB1−^ in G2 phage was higher than TC ^ITGB1+^.

## Discussion

TCs connect with themselves or other cells through three-dimensional networks of TCs-specific telopodes and cell proliferation to maintain the cell–cell communication and microenvironmental stability and accelerate the process of tissue repair [19]. PI3K and TGF family networks in TCs differed from other cells which appear in the lung tissue and PI3K-TGF signal pathways play the critical role in the regulation of TCs proliferation and expression of PI3K isoform genes [5]. The present study furthermore investigates roles of ITGB1 in PI3K-TGF signal pathways-regulated TCs bio-behaviors. Different integrin subunits binding to the same ligand can trigger different signal transduction, and the expression patterns of integrins on cell surface is the key to determine the response of cells to microenvironments to immune and inflammatory regulation [19–21]. Although ITGB1 on alveolar epithelium was found to alleviate epithelial injury and remodeling in acute respiratory distress syndrome [22], profiles and functions of ITGB1 and their family members in lung TCs remain unclear due to the limited source of preliminary lung TCs. The present study firstly reported characters of ITGB family member networks and interactions in the primary mouse lung TCs, highly dependent upon cell types and functions. Of elements in TCs networks and interactions, ITGB4 declined and ITGB1 increased rapidly on day 10. We found that the interaction between PI3K and ITGB1 could complement the mechanisms of TCs proliferation and ITGB1 regulated TC sensitivity to PI3K isoform protein inhibition or PKC inhibition.

ITGB1 plays critical roles in the maintenance of cell proliferation and movement, evidenced by the finding that the over-expression of ITGB1 genes could increase cancer cell proliferation and metastasis which was declined by deletion of ITGB1 gene [23]. TGFβ activation depends upon the cytoplasmic domain integrity of ITGB subunit which connects with actin cytoskeleton or the extracellular environment, e.g. specific binding of ITGαvβ6 and αvβ8 to the arm domain of Pro-TGFβ1 [24]. Our data suggest that ITGB1 play the decisive role in TC survival and proliferation and change TC sensitivity to stimulators or inhibitors. The deletion of ITGB1 increased the sensitivity of TCs to PI3Kp/110α, PI3Kα/δ, PKCβ, or GSK3 inhibitors, indicating that ITGB1 contributes to multiple signal pathways and the interaction between ITGB1 and TGFβ regulates cell fate and proliferation through intracellular PI3K/PKC/GSK pathways. For example, the binding of ITGαvβ6 with TGFβ1 could regulate the production of inflammatory mediators through the activation of HMGB1 in injured epithelial cells, accelerating lung tissue repair and curing [25]. We noticed that the dose of TGFβ1 which did not increase TC^ITGB1+^ proliferation could accelerate TC^ITGB1−^ proliferation, and such effects of TGFβ1 became more obvious when PI3K/PKC/GSK pathways were down-regulated in TC^ITGB1−^ cells, especially GSK3α and β signal pathways.

The present study demonstrates that intracellular ITGB1-TGFβ1-PI3K signal pathways in TCs have the strong compensatory capacity to maintain cell survival and proliferation. It was evidenced by the finding that TGFβ1 increased proliferation increased in TCs with combined inhibition of ITGB1 and one of PI3K/p110α, PI3Kα/δ, PKCβ, or GSK3β signaling. We propose that the hyper-sensitivity of TC^ITGB1−^ with either inhibitor may result from the compensatory mechanisms of other signal pathway function or crosstalk as in other condition [26–28]. The interaction of ITGB-PI3K plays important roles in the maintenance of normal lung cell hemostasis and function and the response to inflammation, though it is unclear in lung TCs. For example, ITGB1 protein could regulate the activation of NFκB and the expression of pro-inflammatory cytokines in alveolar macrophage [20], control intracellular transcription, accelerate cell cycle progression, movement, survival, metabolism, and DNA damage [29]. TGF-β regulates cell fate and plasticity as well as multifunctional cellular responses via mothers against decapentaplegic homolog (SMAD) or non-SMAD pathways or ITGB1 pathway band with fibronectins and tenascin-C PI3K/AKT pathway, as explained in Fig. 7. The conversion from oleate-activated transcription factor (PIP2) to plasma membrane intrinsic protein 3 (PIP3) regulated by PI3K isoform proteins is the central process of the interaction between Akt and GSK or between PKC and Akt directly or indirectly [30–32].

**Fig. 8:**
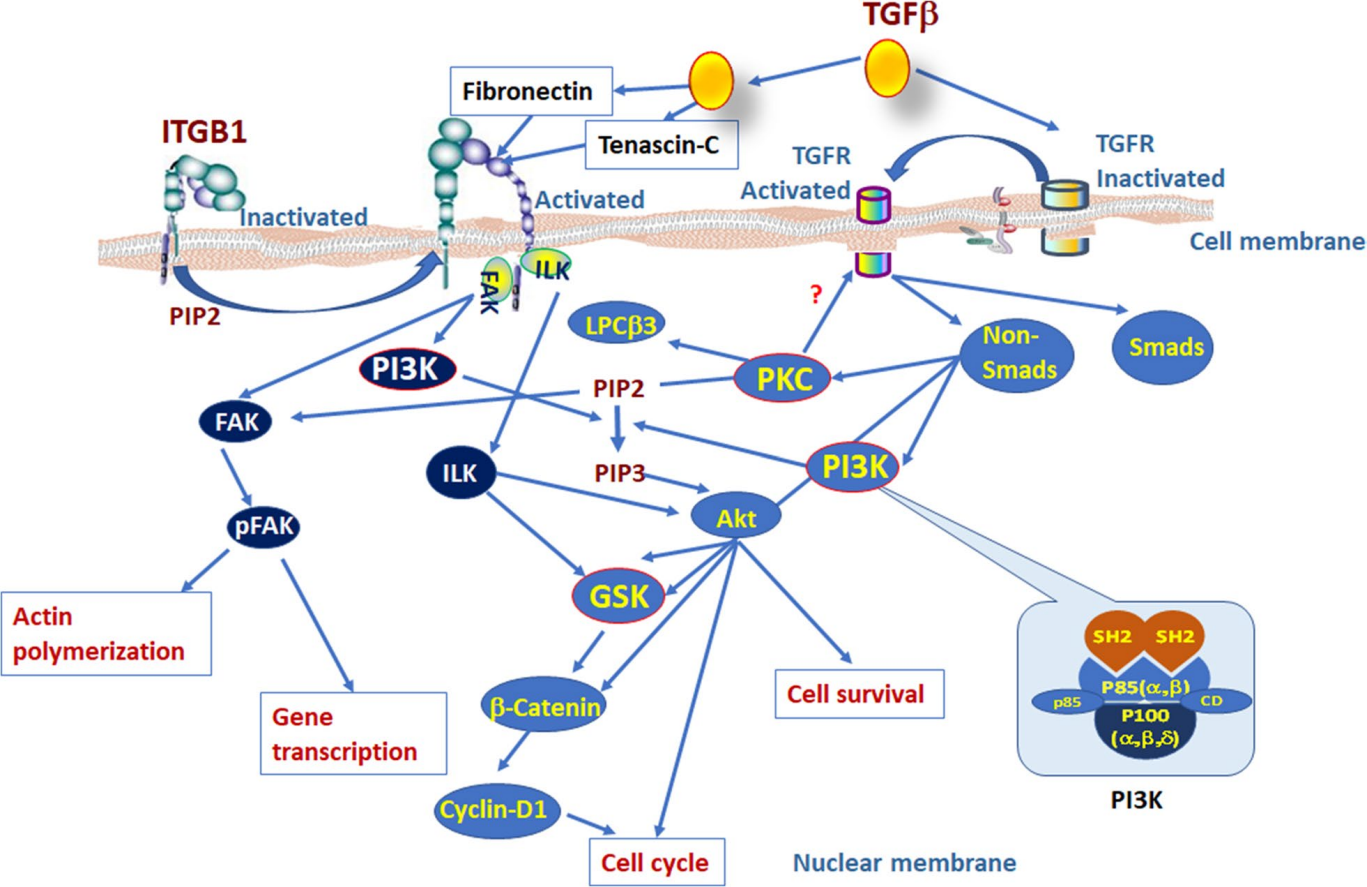
The explanation of TGF-β regulates cell fate and plasticity as well as multifunctional cellular responses via SMADor non-SMAD pathways or ITGB1 pathway band with fibronectins and tenascin-C PI3K/AKT pathway. The molecules with red circles were key signaling pathways in our current study [30–34]

It is also possible that PI3K inhibitors could inhibit the fibrinogen/tenascin-C binding to ITGαIIbβ3 by inducing GTPase-Rap1 or the PI3K p110β and PI3K p110γ directly as downstream integrins by inducing activation of G protein-coupled receptors [30]. Figure 7 demonstrates that integrins regulate the activity of PI3K signaling pathway and cause the accumulation of PIP3. ITGα2β1 was found to activate PI3K p110β, RasGRP2, or GTP binding to Rap1b, to amplify the activation of ITGα2β1signals and forming regulatory feedback loops around the cell membrane [31]. The interaction between ITGB-PI3K could influence the selective bind to ITGαvβ3 receptor to regulate cell cycle, cell proliferation and cell migration [32]. Our results demonstrate that ITGB1 plays the significant role in the regulation of TC proliferation, since the inhibition of PI3Kp110α, PI3Kα/δ, or PKCβ caused the increase in S phase of TCs with ITGB1, while mainly in G1 phase in TCs without ITGB1, different from the inhibition of GSK3β. We hypothesize that PIK3CG gene and protein p110-γ may play the regulatory roles in cell cycle responses to TGF and those inhibitors in the absence of ITGB1. PI3K/Akt plays important roles in the molecular mechanism of cell cycle and survival through the signals of Akt/PKC/nuclear factor kappa-B (NF-κB) and GSK-3 [33] and regulates GSK-3β-dominated downstream survival signaling pathway of serine/threonine kinase [34]. Our results indicate that both TGF-β-band type II and type I serine and threonine kinase receptors (TβRII and TβRI) and ITGB1 can activate PI3K signal pathway either via the switch of the heterotetrameric receptor complex from inactivated to activated TβRII and TβRI or via the switch of the integrin-mediated focal adhesion kinase signaling from inactivated to activated ITGB1. Both switches are required for the activation of class IA PI3K mediated by TGFβ1 [35] and also result in the activation of PI3Kp110α, PI3Kα/δ, PKCβ, or GSK3 (Fig. 8). Molecules and networks in the TGFβ/ITGB1/PI3K pathways can be considered as TC-specific functional biomarkers and therapeutic targets like others [36–40].

In conclusion, we found the characters and interactions of ITG or PKC family member networks in primary mouse lung TCs and defined their specificity and dynamic alternations, different from other cells which often appear in the lung tissue. The deletion of ITGB1 changed TCs sensitivity to treatment with multifunctional cytokines or signal pathway inhibitors. The compensatory mechanisms may occur among TGFβ1-induced PI3Kp110α, PI3Kα/δ, PKCβ, or GSK3 when ITGB1 gene was deleted, leading to alterations of TCs cell cycle and proliferation. Of those PI3K isoform protein genes, mRNA expression of *PIK3CG* altered with ITGB1-negative TC cycle and proliferation. Such compensatory mechanism can provide insights for understanding molecular mechanisms by which TCs have strong capacity of proliferation and repair and of cell–cell communication and for investigating the potential roles of TCs in the development of drug resistance.

## Supplementary information

**Additional file 1: Figure S1.** The characters of GSK networks and interactions in mouse lung primary TCs cultured for 5 days (TC 5) and 10 days (TC 10) were compared with other tissue cells, e.g. airway basal cells (Basal cells), proximal airway cells (Duct cells).

**Additional file 2: Figure S2.** The effects of ITGB1 on the cell proliferation and movement curves of TCs treated with TGFβ1. A. Analysis of TC ^ITGB1+^ or TC^ITGB1−^ cell proliferation curves with the treatment of TGFβ1. B. Analysis of TC ^ITGB1+^ or TC^ITGB1−^ cell movement curves with the treatment of TGFβ1, n = 6–8, *stand for *p* values less than 0.05, as compared with TC ^ITGB1+^; ^*#*^ stand for *p* values less than 0.05, respectively, as compared with NC.

**Additional file 3: Figure S3.** The effects of ITGB1on the cell differentiation curve of TCs treated with TGFβ1 and PI3Kp110α, PI3Kα/δ, PKCβ, GSK3 inhibitors, respectively, n = 6–8.

**Additional file 4: Figure S4.** The effects of ITGB1on the cell death curve of TCs treated with TGFβ1 and PI3Kp110α, PI3Kα/δ, PKCβ, GSK3 inhibitors, respectively, n = 6–8, *stand for *p* values less than 0.05, as compared with TC ^ITGB1+^ treated with TGFβ1 and PI3K inhibitors.

**Additional file 5: Figure S5.** Cell bio-behaviors of TC ^ITGB1+^ or TC^ITGB1−^ treated with TGFβ1 and PI3Kp110α, PI3Kα/δ, PKCβ, GSK3 inhibitors, respectively, n= 6–8.

## Data Availability

Not applicable.

## Abbreviations

TCs: telocytes
Tps: telopodes
SV40: simian vacuolating virus 40 small and large T antigen
TGF-β: transforming growth factor-β
SPSS: Statistical Package for the Social Sciences
ANOVA: one‑way analysis of variance
PI3K: phosphatidylinositol 3′ –kinase
GSK-3: glycogen synthasc kinase-3
mTOR: Mammalian target of rapamycin
GSK-3: glycogen synthase kinase-3
ITG: integrins
ITGB1: integrin beta1
PKC: protein kinase C
DEN: differential network model
CDK: cyclin-dependent kinases
TC^ITGB1−^: TCs knocked out of ITGB1
TC 5: mouse lung primary TCs cultured for 5 days
TC 10: mouse lung primary TCs cultured for 10 days
Fbs: fibroblasts
MSCs: mesenchymal stem cells
ATII: alveolar type II cells
ABCs: airway basal cells
PACs: proximal airway cells
CD8^+^ T-BL: CD8^+^ T cells come from bronchial lymph nodes
CD8^+^ T-LL: CD8^+^ T cells come from lung
ARDS: acute respiratory distress syndrome
PIP2: oleate-activated transcription factor
PIP3: plasma membrane intrinsic protein 3
SMAD: mothers against decapentaplegic homolog
NF-κB: nuclear factor kappa-B
