# Supplementary figures and images for: Roles of transforming growth factor-β and phosphatidylinositol 3-kinase isoforms in integrin β1-mediated bio-behaviors of mouse lung telocytes
Source: J Transl Med. 2019 Dec 30;17:431. doi: 10.1186/s12967-019-02181-2 (PMC6936066; doi:10.1186/s12967-019-02181-2)

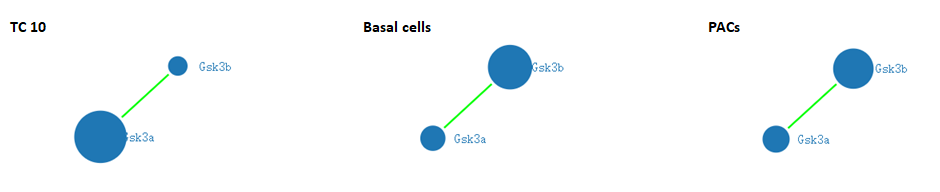

Supplement: Supplementary file 1 — Additional file 1: Figure S1. The characters of GSK networks and interactions in mouse lung primary TCs cultured for 5 days (TC 5) and 10 days (TC 10) were compared with other tissue cells, e.g. airway basal cells (Basal cells), proximal airway cells (Duct cells). [file 12967_2019_2181_MOESM1_ESM.png]

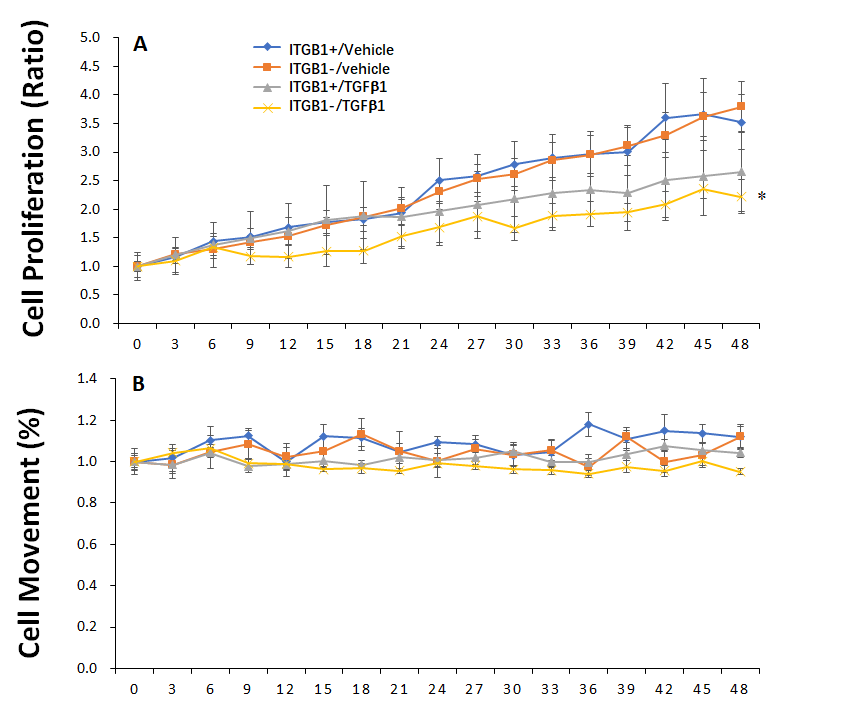

Supplement: Supplementary file 2 — Additional file 2: Figure S2. The effects of ITGB1 on the cell proliferation and movement curves of TCs treated with TGFβ1. A. Analysis of TC ITGB1+ or TCITGB1− cell proliferation curves with the treatment of TGFβ1. B. Analysis of TC ITGB1+ or TCITGB1− cell movement curves with the treatment of TGFβ1, n = 6–8, *stand for p values less than 0.05, as compared with TC ITGB1+; # stand for p values less than 0.05, respectively, as compared with NC. [file 12967_2019_2181_MOESM2_ESM.png]

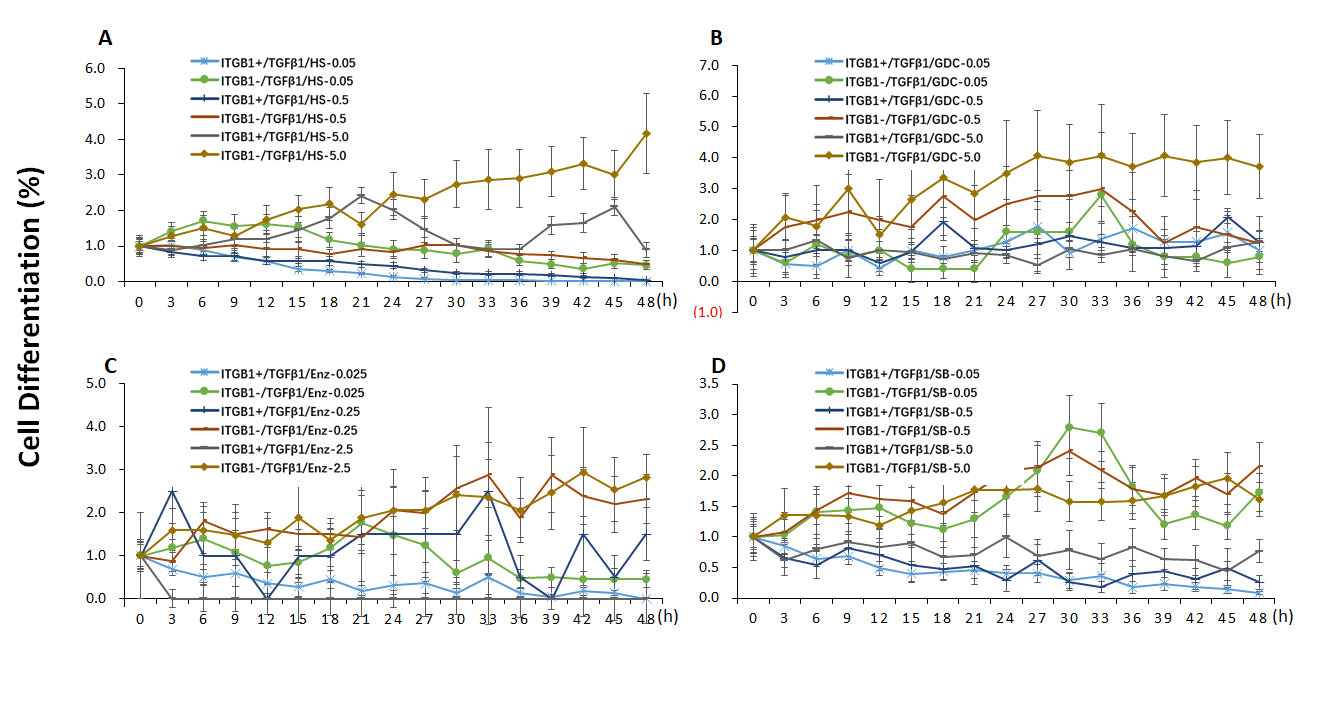

Supplement: Supplementary file 3 — Additional file 3: Figure S3. The effects of ITGB1on the cell differentiation curve of TCs treated with TGFβ1 and PI3Kp110α, PI3Kα/δ, PKCβ, GSK3 inhibitors, respectively, n = 6–8. [file 12967_2019_2181_MOESM3_ESM.png]

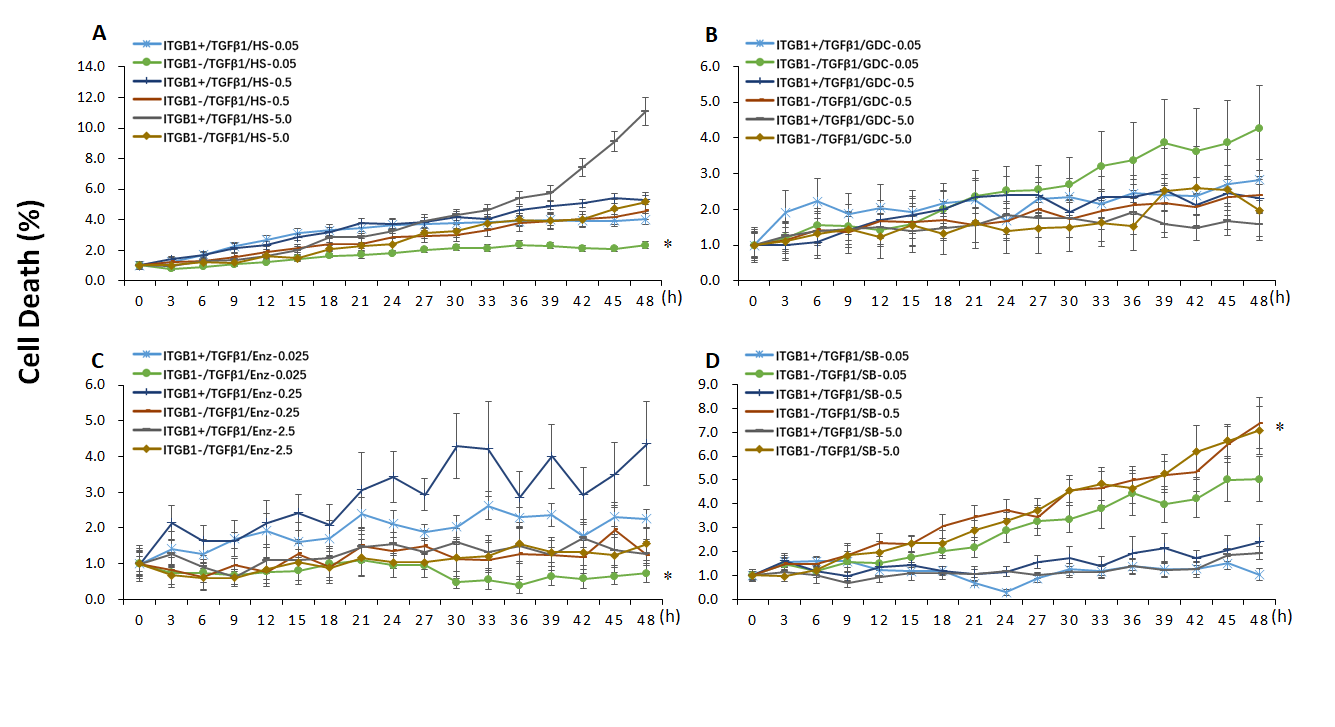

Supplement: Supplementary file 4 — Additional file 4: Figure S4. The effects of ITGB1on the cell death curve of TCs treated with TGFβ1 and PI3Kp110α, PI3Kα/δ, PKCβ, GSK3 inhibitors, respectively, n = 6–8, *stand for p values less than 0.05, as compared with TC ITGB1+ treated with TGFβ1 and PI3K inhibitors. [file 12967_2019_2181_MOESM4_ESM.png]

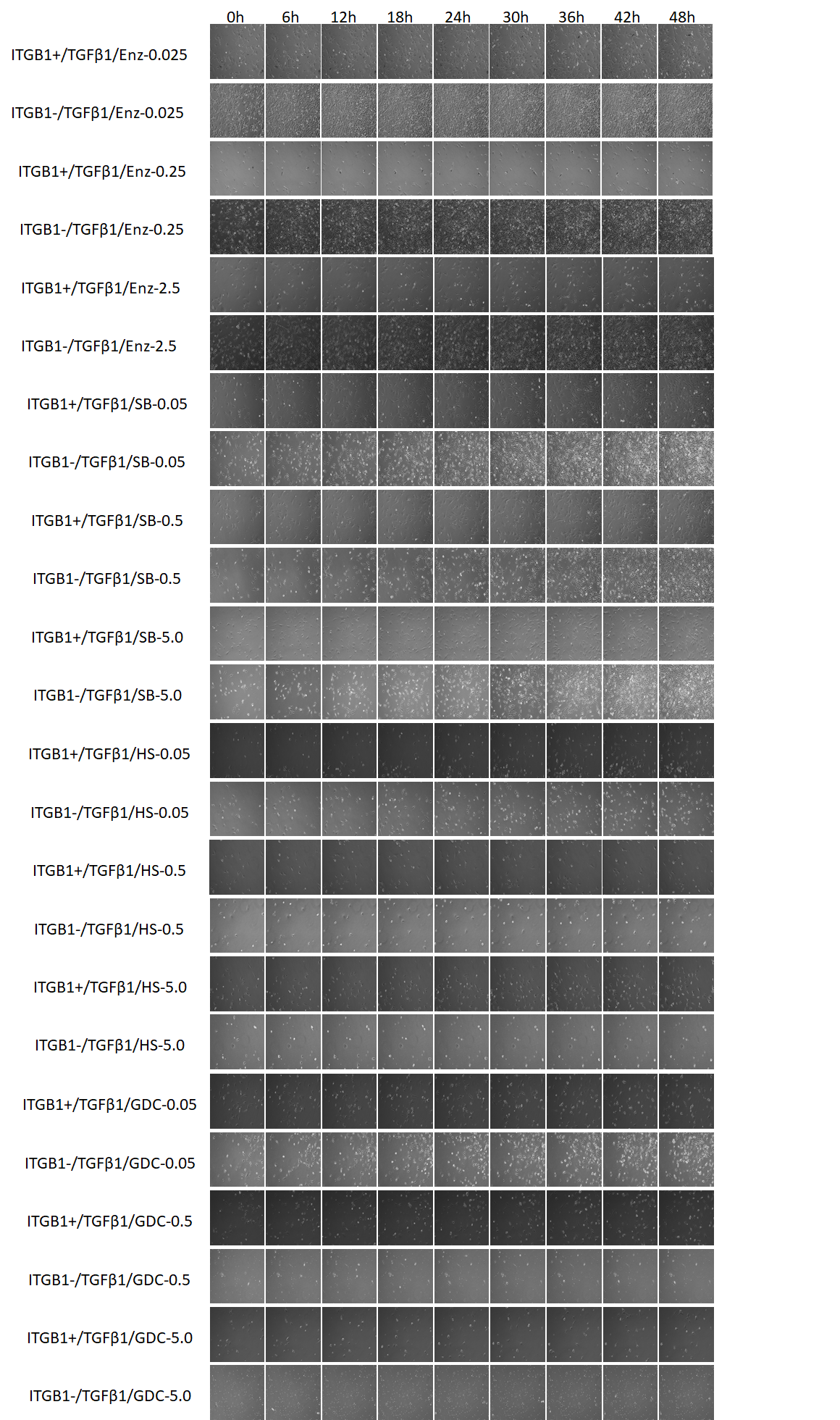

Supplement: Supplementary file 5 — Additional file 5: Figure S5. Cell bio-behaviors of TC ITGB1+ or TCITGB1− treated with TGFβ1 and PI3Kp110α, PI3Kα/δ, PKCβ, GSK3 inhibitors, respectively, n= 6–8. [file 12967_2019_2181_MOESM5_ESM.png]
